# Ac2-26, an Annexin A1 Peptide, Attenuates Ischemia-Reperfusion-Induced Acute Lung Injury

**DOI:** 10.3390/ijms18081771

**Published:** 2017-08-15

**Authors:** Wen-I Liao, Shu-Yu Wu, Geng-Chin Wu, Hsin-Ping Pao, Shih-En Tang, Kun-Lun Huang, Shi-Jye Chu

**Affiliations:** 1 The Graduate Institute of Medical Sciences, National Defense Medical Center, Taipei 114, Taiwan; qqww0139@yahoo.com.tw (W.-I.L.); simple5252@hotmail.com (H.-P.P.); 2 Department of Emergency Medicine, Tri-Service General Hospital, National Defense Medical Center, Taipei 114, Taiwan; 3 Institute of Aerospace and Undersea Medicine, National Defense Medical Center, Taipei 114, Taiwan; shuyu0321@gmail.com; 4 Department of Internal Medicine, Taoyuan Armed Forces General Hospital, Taoyuan 325, Taiwan; medicine804h@yahoo.com.tw; 5 Department of Internal Medicine, Tri-Service General Hospital, National Defense Medical Center, Taipei 114, Taiwan; t4n000@yahoo.com.tw

**Keywords:** acute lung injury, ischemia-reperfusion, Ac2-26, annexin A1, nuclear factor-κB

## Abstract

Annexin A1 (AnxA1) is an endogenous protein that modulates anti-inflammatory processes, and its therapeutic potential has been reported in a range of inflammatory diseases. The effect of AnxA1 on ischemia-reperfusion (IR)-induced lung injury has not been examined. In this study, isolated, perfused rat lungs were subjected to IR lung injury induced by ischemia for 40 min, followed by reperfusion for 60 min. The rat lungs were randomly treated with vehicle (phosphate-buffered saline), and Ac2-26 (an active N-terminal peptide of AnxA1) with or without an *N*-formyl peptide receptor (FPR) antagonist N-Boc-Phe-Leu-Phe-Leu-Phe (Boc2). An in vitro study of the effects of Ac2-26 on human alveolar epithelial cells subjected to hypoxia-reoxygenation was also investigated. Administration of Ac2-26 in IR lung injury produced a significant attenuation of lung edema, pro-inflammatory cytokine production recovered in bronchoalveolar lavage fluid, oxidative stress, apoptosis, neutrophil infiltration, and lung tissue injury. Ac2-26 also decreased AnxA1 protein expression, inhibited the activation of nuclear factor-κB and mitogen-activated protein kinase pathways in the injured lung tissue. Finally, treatment with Boc2 abolished the protective action of Ac2-26. The results indicated that Ac2-26 had a protective effect against acute lung injury induced by IR, which may be via the activation of the FPR.

## 1. Introduction

The annexin A1 (AnxA1) protein is an important endogenous inhibitory regulator of inflammation that is turned on in response to tissue injury. The AnxA1 protein is highly expressed in respiratory, renal, brain, and cardiac tissues, as well as in a variety of cells such as neutrophils, fibroblasts, macrophages, and epithelial cells [1]. This expression is markedly upregulated by ischemia-reperfusion (IR), oxidative stress, and glucocorticoids [2]. Evidence indicates that AnxA1 mediates most of its biologic functions via interaction with an *N*-formyl peptide receptor (FPR) [2,3,4]. AnxA1 suppresses neutrophil adhesion and migration through the inflammatory post-capillary endothelium, attenuates neutrophil recruitment in the inflamed tissue, separates adhered leukocytes from the endothelium, and inhibits phospholipase A2 activity [5]. Furthermore, AnxA1 has been shown to suppress nuclear factor (NF)-κB activation and pro-inflammatory cytokine production [2]. Treatment with AnxA1 (or the peptide Ac2-26 derived from its N-terminal end comprising residues 2–26 of AnxA1), which maintains the anti-inflammatory activity of AnxA1 protein, has been reported to provide protection against a range of animal models of inflammatory diseases, such as endotoxemia, colitis, arthritis, and ischemia-reperfusion (IR) injury in the heart, liver, kidney, and brain [2]. In an endotoxemia model, AnxA1-deficient mice showed a marked increase in the production of the pro-inflammatory cytokines interleukin (IL)-6 and tumor necrosis factor (TNF)-α, as well as exacerbated organ damage and mortality [2,6]. Therefore, therapies directly targeting the AnxA1 signaling pathway could be a novel therapeutic approach to treat various inflammatory diseases [1].

IR-induced acute lung injury and acute respiratory distress syndrome (ALI/ARDS) may develop in various clinical conditions, including lung transplantation, hemorrhagic shock, septic shock, cardiopulmonary bypass, resuscitation for cardiac arrest, and pulmonary embolism. It likely contributes to long-term tissue damage associated with dysfunction and mortality [7]. ALI/ARDS is characterized by alveolar damage, lung inflammation, pro-inflammatory cytokine production, neutrophil infiltration and accumulation, and microvascular injury leading to lung edema [7,8]. Our previous studies of IR lung injuries demonstrated that the inflammatory response played a critical role in the pathogenesis of such injuries [9], where it is tightly regulated by opposite-regulators of endogenous anti-inflammatory and pro-resolving mediators [1,5]. These anti-inflammatory mediators may act at different phases of the inflammatory response [1]. Recently, AnxA1 displayed potent anti-inflammatory effects in lung inflammation induced by lipopolysaccharide (LPS) and intestinal IR in mice [10,11]. Currently, the role of AnxA1 in IR-induced lung injury has not yet been determined. Therefore, we investigated whether the Anx-1 N-terminal peptide Ac2-26 could protect rats from experimental IR-induced lung injury. We also investigated the mechanism underlying lung protection, including the involvement of FPR, using the nonselective FPR1/FPR2 antagonist N-Boc-Phe-Leu-Phe-Leu-Phe (Boc2).

## 2. Results

A diagram of the experimental protocol is provided in the Supplementary Material (Figure S1).

### 2.1. Effect of Ac-AMVSEFLKQAWFIENEEQEYVQTVK (Ac2-26) on Indices of Lung Edema

**Figure 1:**
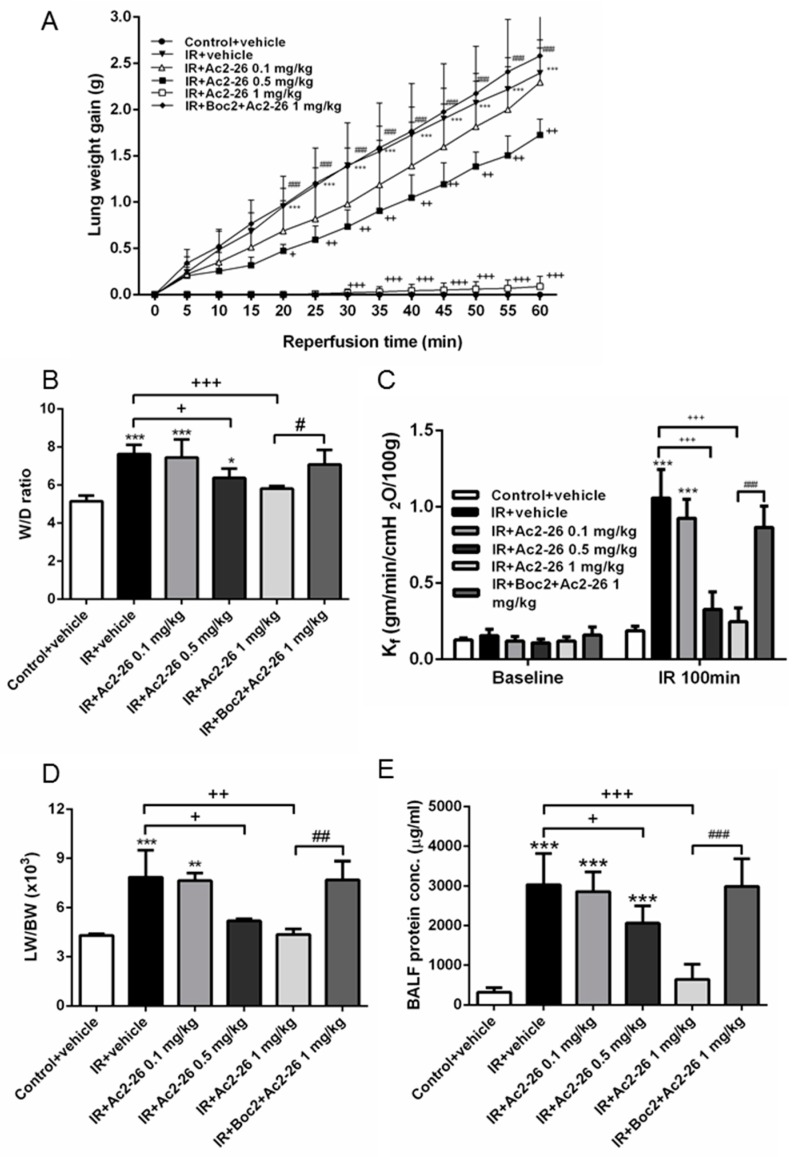
Effect of Ac-AMVSEFLKQAWFIENEEQEYVQTVK (Ac2-26) and N-Boc-Phe-Leu-Phe-Leu-Phe (Boc2) on pulmonary edema. Lung weight gain (**A**); wet/dry (W/D) weight ratio (**B**); vascular filtration coefficient (K_f_) (**C**); lung weight/body weight (LW/BW) (**D**); and protein concentration in bronchoalveolar lavage fluid (BALF) (**E**) increased significantly in the ischemia-reperfusion (IR) group. The increase in these parameters was significantly attenuated by treatment with Ac2-26. The protective effect of Ac2-26 was abrogated by the addition of Boc2. Data are expressed as means ± standard deviation (SD) (*n* = 6 per group). * *p* < 0.05, ** *p* < 0.01, *** *p* < 0.001, compared with the control group; ^+^ *p* < 0.05, ^++^ *p* < 0.01, ^+++^ *p* < 0.001, compared with the IR group; ^#^ *p* < 0.05, ^##^ *p* < 0.01, ^###^ *p* < 0.001, compared with the IR + AC2-26 1 mg/kg group.

The increase in lung weight gain in the IR group was attenuated in a dose-dependent manner by the Ac2-26 treatment (Figure 1A). Furthermore, Ac2-26 treatment in the IR group also reduced these increases in wet to dry (W/D) weight ratio, vascular filtration coefficient (K_f_), lung weight/body weight (LW/BW) ratio, and protein concentrations in the bronchoalveolar lavage fluid (BALF) (*p* < 0.05; Figure 1B–E). However, the addition of Boc2 abolished the protective effects of Ac2-26. Moreover, during the 100-min observation period, the pulmonary arterial pressure (PAP) in the control group remained constant. Treatment with Ac2-26 in the IR group diminished the increase in the rise of PAP at 60 min after reperfusion in a dose-dependent fashion. The protective effect of Ac2-26 was significantly reversed when Boc2 was added (*p* < 0.05; Supplementary Material, Figure S2).

### 2.2. Effect of Ac2-26 on AnxA1 Protein Expression in Lung Tissue

**Figure 2:**
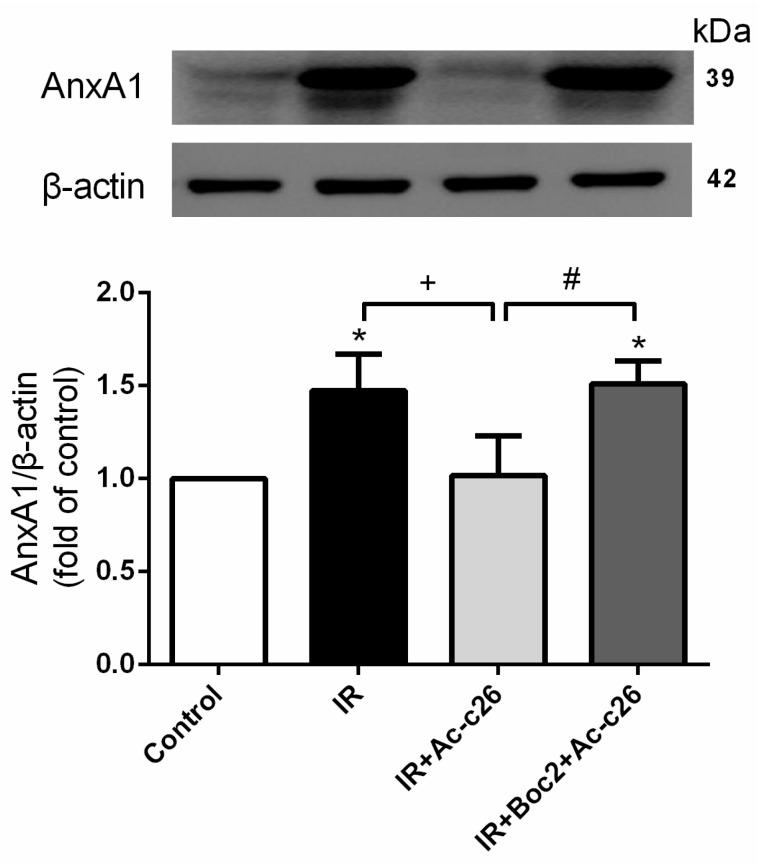
Effect of Ac2-26 and Boc2 on AnxA1 protein expression in lung tissue. IR significantly increased AnxA1 protein expression in the lung tissue. Ac2-26 significantly decreased AnxA1 protein expression. The addition of Boc2 reversed the effect of Ac2-26. Data are expressed as means ± SD (*n* = 6 per group). * *p* < 0.05, compared with the control group; ^+^ *p* < 0.05, compared with the IR group; and ^#^ *p* < 0.05, compared with the IR + Ac2-26 group.

IR significantly increased AnxA1 protein when compared with the control group (*p* < 0.05; Figure 2). Furthermore, Ac2-26 (1 mg/kg) treatment significantly decreased AnxA1 protein expression in comparison with the IR group. When Boc2 was added in the Ac2-26 (1 mg/kg) treatment group, AnxA1 protein expression was significantly increased when compared with the IR+Ac2-26 group (*p* < 0.05; Figure 2).

### 2.3. Effect of Ac2-26 on Cytokine-Induced Neutrophil Chemoattractant-1 (CINC-1) and TNF-α Concentrations in Bronchoalveolar Lavage Fluid (BALF)

**Figure 3:**
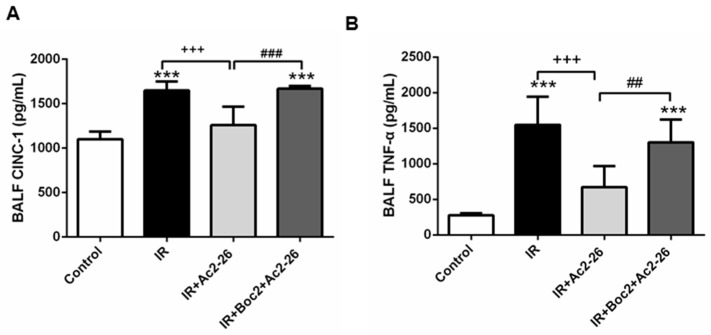
Effect of Ac2-26 and Boc2 on cytokine-induced neutrophil chemoattractant-1 (CINC-1) and tumor necrosis factor (TNF)-α levels in BALF. CINC-1 (**A**); and TNF-α (**B**) levels in the BALF increased significantly in the IR group. The increases in the BALF were significantly attenuated by treatment with Ac2-26. The protective effect of Ac2-26 was abrogated by the addition of Boc2. Data are expressed as means ± SD (*n* = 6 per group). *** *p* < 0.001, compared with the control group; ^+++^ *p* < 0.001, compared with the IR group; and ^##^ *p* < 0.01, ^###^ *p* < 0.001, compared with the IR + Ac2-26 group.

Ac2-26 (1 mg/kg) treatment significantly decreased the concentrations of cytokine-induced neutrophil chemoattractant-1 (CINC-1) and TNF-α in the IR group (*p* < 0.001; Figure 3). However, the addition of Boc2 blocked the protective effects of Ac2-26.

### 2.4. Effect of Ac2-26 on Carbonyl Content, Malondialdehyde (MDA) Level, and Myeloperoxidase (MPO)-Positive Cells in Lung Tissue

**Figure 4:**
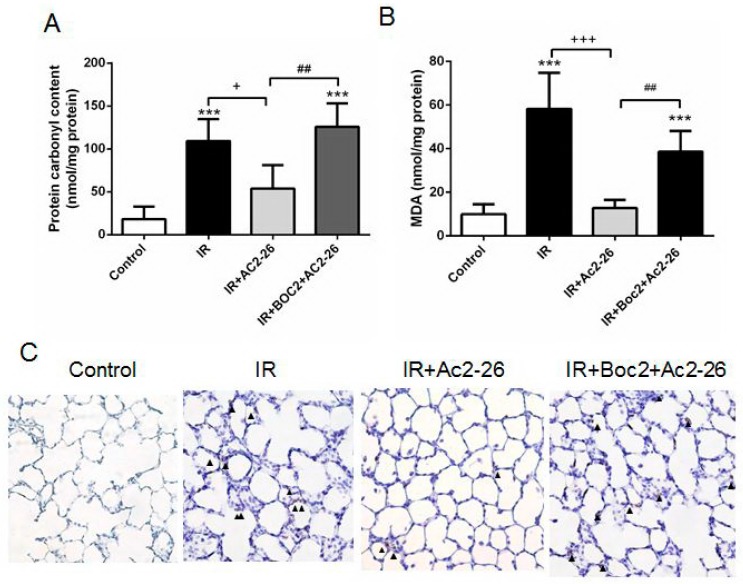
Effect of Ac2-26 and Boc2 on protein carbonyl contents, malondialdehyde (MDA) levels, and myeloperoxidase (MPO)-positive cells in lung tissue. Carbonyl contents (**A**); MDA levels (**B**); and MPO-positive cells (**C**) in lung tissue significantly increased in the IR group. Ac2-26 treatment significantly attenuated these increases. The protective effect of Ac2-26 was abrogated by the addition of Boc2; (**C**) Immunohistochemistry for MPO in the lung (arrowheads indicated MPO-positive cells) (200× magnification). Data are expressed as means ± SD (*n* = 6 per group). *** *p* < 0.001, compared with the control group; ^+^ *p* < 0.05, ^+++^ *p* < 0.001, compared with the IR group; and ^##^ *p* < 0.01, compared with the IR + Ac2-26 group.

Malondialdehyde (MDA) is a frequently used biomarker of oxidative stress. The protein carbonyl content represents a maker of oxidative damage to the proteins in the lung tissue based on a reaction with dinitrophenylhydrazine. Myeloperoxidase (MPO) is a peroxidase enzyme expressed largely in the neutrophil granules. The neutrophil infiltration in the lung alveolar space could be semi-quantitatively confirmed by increasing the MPO-positive cells. Treatment with Ac2-26 (1 mg/kg) significantly attenuated the increases in protein carbonyl contents, MDA levels, and the numbers of MPO-positive cells in the lung tissue of the IR group (Figure 4A–C). However, the protective effects of Ac2-26 were blocked by treatment with Boc2. 

### 2.5. Effect of Ac2-26 on Lung Pathology

**Figure 5:**
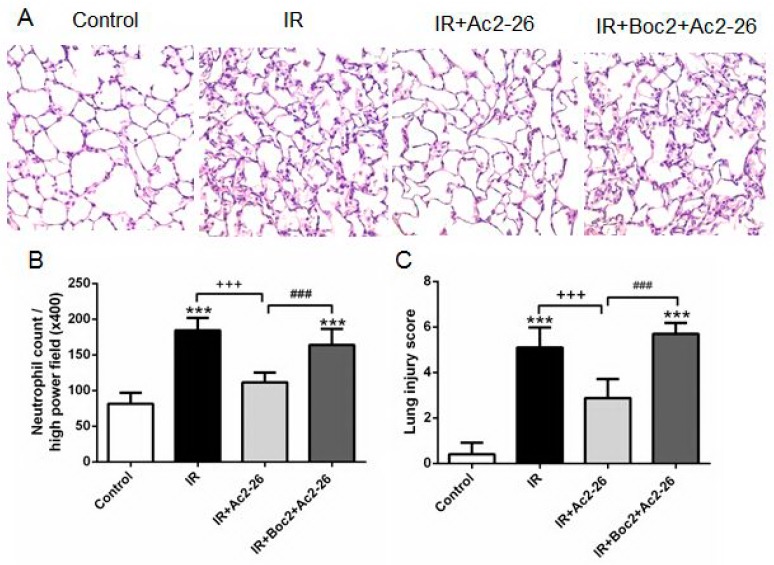
Effect of Ac2-26 and Boc2 on lung pathology. As shown by a representative micrograph of lung tissue (400× magnification) (**A**); neutrophil infiltration and septal edema were increased in the IR group. Ac2-26 treatment improved the histopathological changes, but the improvement was abolished by the addition of Boc2. Ac2-26 treatment significantly attenuated the numbers of neutrophils per high power field (400× magnification) (**B**); and the lung injury scores (**C**), but these improvements were abolished by the addition of Boc2. Data are expressed as means ± SD (*n* = 6 per group). *** *p* < 0.001, compared with the control group; ^+++^ *p* < 0.001, compared with the IR group; and ^###^ *p* < 0.001, compared with the IR + Ac2-26 group.

Compared with the control group, the IR group represents the obvious thickening of the alveolar walls and leukocyte infiltrates in the lung histology, which revealed morphological evidence of acute lung injury (Figure 5A). The Ac2-26-treated group (1 mg/kg) showed significantly decreased neutrophil infiltration (Figure 5B) and lung injury scores (Figure 5C). In contrast, the protective effects of Ac2-26 were eliminated by the addition of Boc2.

### 2.6. Effect of Ac2-26 on Claudin-3, Occludin, and Zonula Occludens-1 (ZO-1) Protein Expression in Lung Tissue

**Figure 6:**
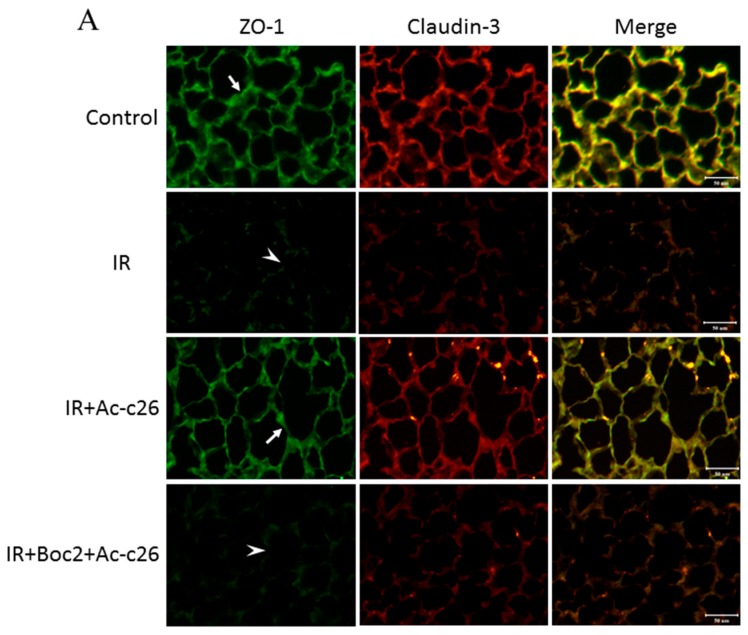

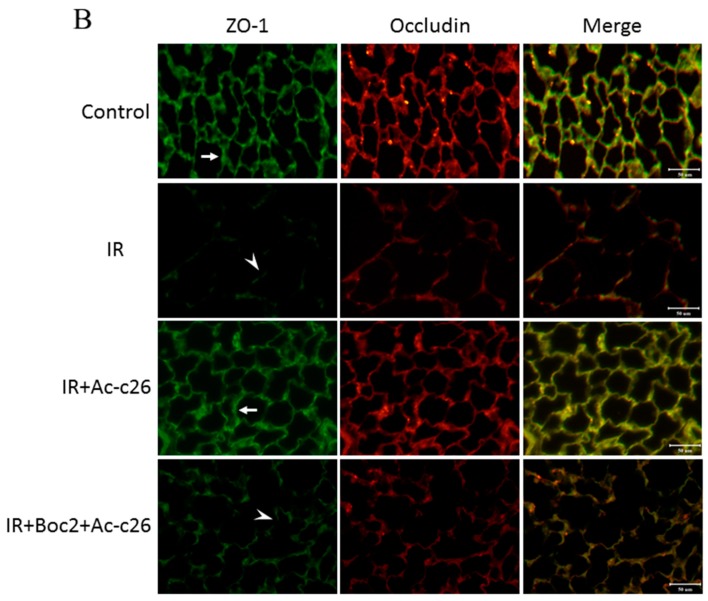
Effect of Ac2-26 on claudin-3, occludin, and zonula occludens-1 (ZO-1) protein expression in lung tissue. The control group had a strong linear staining of a tight junction protein (indicated with an arrow) (**A**) claudin-3 (green); (**B**) occludin (green), and ZO-1 (red). In contrast, the IR group showed a defragmented and low-intensity staining in the alveolar septa (indicated with an arrowhead). Ac2-26 treatment revealed only partial changes in the linear staining of claudin-3, occludin, and ZO-1 in the IR group, but these protective effects were abolished by the addition of Boc2. Scale bar: 50 µm.

The tight junction protein of the alveolar epithelial cells, including claudin-3 and occludin, maintained the integrity of the permeability barrier of the alveolar walls. The scaffolding protein as Zonula Occludens-1 (ZO-1) mediated signal transduction via interaction with claudin-3 and occludin. The lung tissue in the control group had a strong linear staining of claudin-3, occludin, and ZO-1. In contrast, the IR group showed a defragmented and low-intensity staining in the alveolar walls. Furthermore, Ac2-26 (1 mg/kg) treatment revealed only partial changes in the linear staining of claudin-3, occludin and ZO-1 in the IR group. However, the protective effects of Ac2-26 were reduced by the addition of Boc2 (Figure 6A,B).

### 2.7. Effects of Ac2-26 on B-Cell Lymphoma (Bcl)-2 and Caspase-3 Protein Expression in Lung Tissue

**Figure 7:**
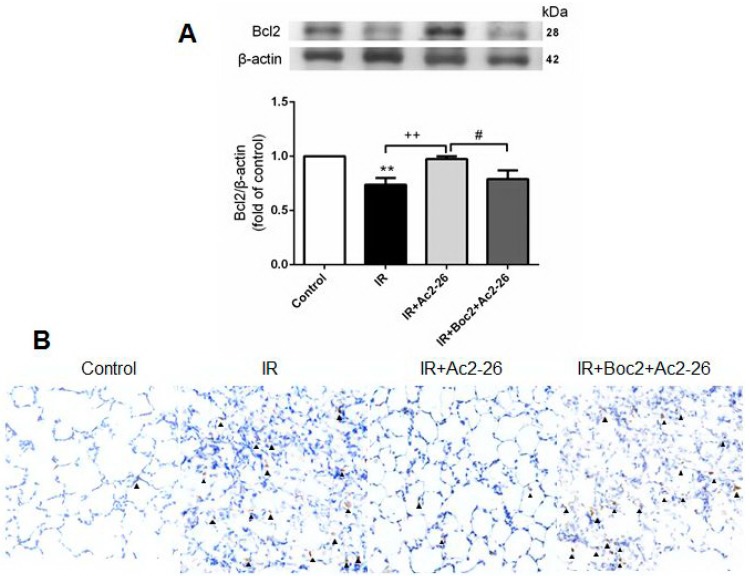
Effect of Ac2-26 and Boc2 on the expression of B-cell lymphoma (Bcl)-2 and caspase-3 in lung tissue. (**A**) Western blot analysis of Bcl-2 protein in the lung tissue. β-actin served as a loading control for cytoplasmic proteins. Representative blots are shown; (**B**) Immunohistochemistry for active caspase-3 in the lung (indicated with an arrowhead) (200× magnification). IR significantly decreased Bcl-2 protein expression and induced caspase-3 activation in the lung tissue. Ac2-26 treatment significantly increased Bcl-2 protein expression and attenuated the signals for active caspase-3 in the IR group. When Boc2 was added, the protective effect was blocked. Representative blots are shown. Data are expressed as means ± SD (*n* = 6 per group). ** *p* < 0.01, compared with the control group; ^++^ *p* < 0.01, compared with the IR group; and ^#^ *p* < 0.05, compared with the IR + Ac2-26 group.

Bcl-2 inhibited the actions of pro-apoptotic proteins and can protect the cell from apoptosis. In contrast, caspase-3 was a frequently activated death protease which represented the hallmarks of apoptosis. Ac2-26 (1 mg/kg) treatment significantly increased Bcl-2 protein contents and decreased the number of activated caspase-3-immunolabeled cells in the IR group. However, these protective effects were blocked by the addition of Boc2 (Figure 7A,B).

### 2.8. Effect of Ac2-26 on the Mitogen-Activated Protein Kinase (MAPK) Signaling Pathway and Mitogen-Activated Protein Kinase Phoshphotases-1 (MKP-1) Induction in Lung Tissue

**Figure 8:**
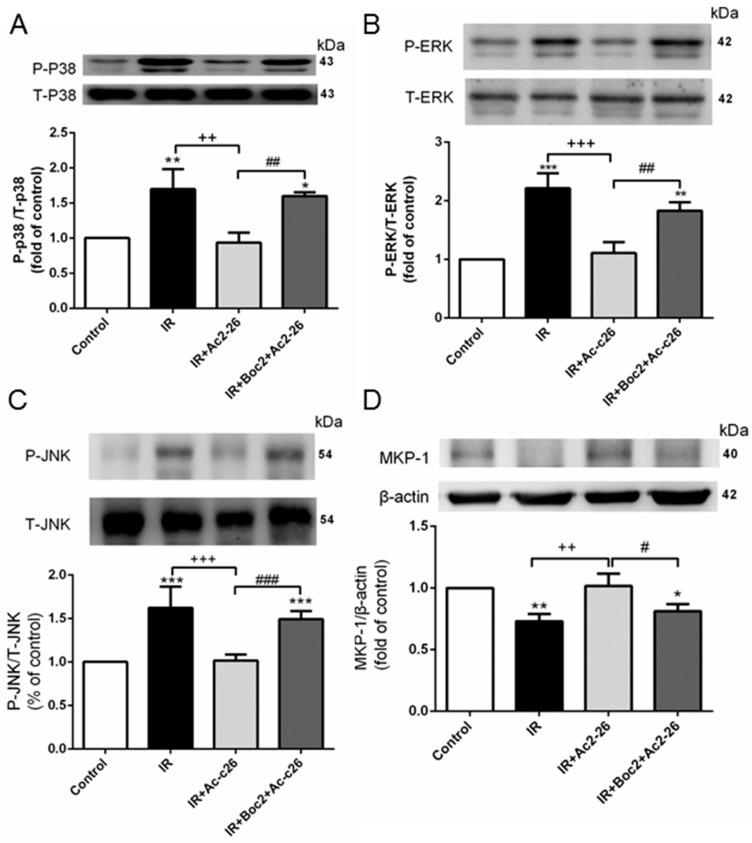
Effect of Ac2-26 and Boc2 on mitogen-activated protein kinase (MAPK) and mitogen-activated protein kinase phoshphotases-1 (MKP-1) expressions in lung tissue. The phosphorylation of p38 protein kinase (p38) (**A**); extracellular signal-related protein kinase 1/2 (ERK) (**B**); and c-Jun N-terminal kinase (JNK) (**C**) was enhanced in the IR group. The effect was attenuated by Ac2-26 treatment. In contrast, the expression of MKP-1 protein (**D**) was decreased in the IR group, but reversed by Ac2-26 treatment. When Boc2 was added, the protective effect of Ac2-26 was blocked. β-actin served as loading controls. An example of a blot is shown. All data are shown as means ± SD (*n* = 6 per group). * *p* < 0.05, ** *p* < 0.01, *** *p* < 0.001, compared with the control group; ^++^ *p* < 0.01, ^+++^ *p* < 0.001, compared with the IR group; and ^#^ *p* < 0.05, ^##^ *p* < 0.01, ^###^ *p* < 0.001, compared with the IR + Ac2-26 group.

The mitogen-activated protein kinase (MAPK) pathways containing three sequentially key protein kinases, including p38 protein kinase (p38), c-Jun N-terminal kinase (JNK), and extracellular signal-related protein kinase 1/2 (ERK), were significantly activated by IR in the lung tissue. These activations were reduced by the administration of Ac2-26 (1 mg/kg, Figure 8A–C). In contrast, IR significantly attenuated the protein expression of MKP-1 in the lung tissue as compared with the control group, which was significantly elevated upon Ac2-26 treatment (Figure 8D). The protective effects of Ac2-26 were reduced by the addition of Boc2.

### 2.9. Effect of Ac2-26 on the Nuclear Factor (NF)-κB Signaling Pathway

**Figure 9:**
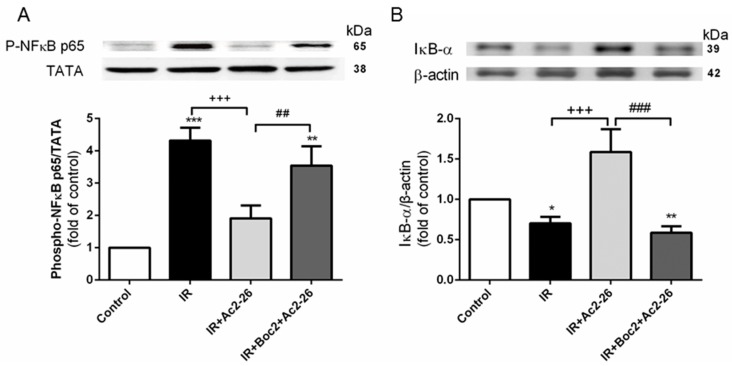
Effect of Ac2-26 and Boc2 on the NF-κB signaling pathway in lung tissues. Ac2-26 reduced nuclear phosphorylated NF-κB p65 levels (**A**) and increased the inhibitor of NF-κB (IκB)-α levels (**B**) in IR-induced lung injury. The addition of Boc2 attenuated the protective effect of Ac2-26. TATA and β-actin served as loading controls for nuclear and cytoplasmic proteins, respectively. Representative blots are shown. Data are expressed as means ± SD (*n* = 6 per group). * *p* < 0.05, ** *p* < 0.01, *** *p* < 0.001, compared with the control group; ^+++^ *p* < 0.001, compared with the IR-vehicle group; and ^##^ *p* < 0.01, ^###^ *p* < 0.001, compared with the IR+Ac2-26 group.

The level of phosphorylated NF-κB p65 in the nucleolus was significantly increased (Figure 9A), whereas the IκB-α in the cytoplasm was significantly decreased in the IR group when compared to the control group (Figure 9B). Ac2-26 (1 mg/kg) treatment significantly increased IκB-α levels and attenuated NF-κB p65 levels. The addition of Boc2 abolished the protective effects of Ac2-26.

### 2.10. Effect of Ac2-26 in A549 Epithelial Cells Subjected to Hypoxia-Reoxygenation (H/R)

**Figure 10:**
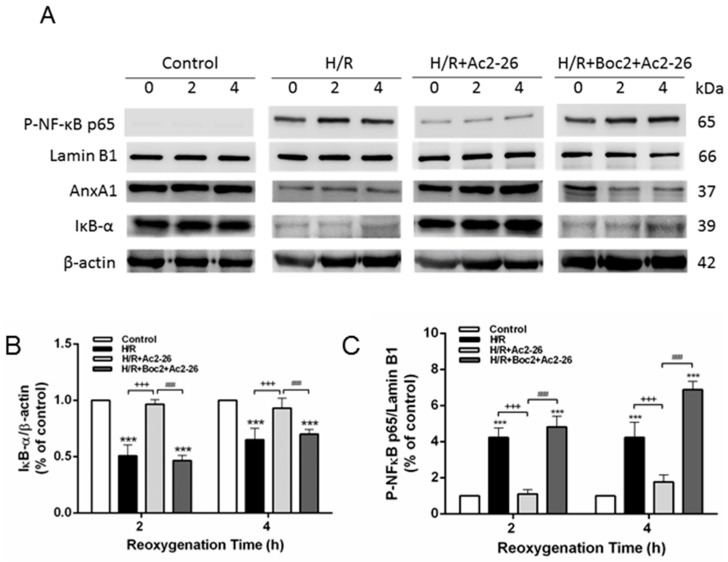

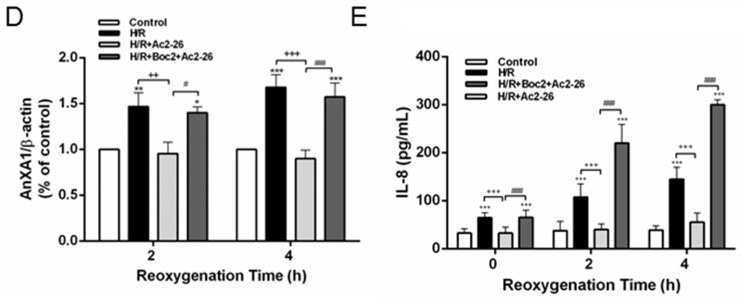
Effect of Ac2-26 and Boc2 in A549 cells subjected to hypoxia-reoxygenation (H/R). Ac2-26 significantly attenuated increased AnxA1 protein expression induced by H/R in A549 cells (**A**). Ac2-26 significantly reduced the increase of phosphorylated NF-κB p65 in the nucleolus, degradation of IκB-α (**A**), and IL-8 production at 2 and 4 h in A549 cells exposed to H/R (**E**). The addition of Boc2 blocked the effect of Ac2-26. A representative blot is shown (**B**–**D**). Lamin B1 served as loading control for nuclear protein. Data are expressed as means ± SD (*n* = 6). * *p* < 0.05, ** *p* < 0.01, *** *p* < 0.001 compared with the control group; ^++^ *p* < 0.05, ^+++^ *p* < 0.001, compared with the H/R group; and ^#^ *p* < 0.05, ^###^ *p* < 0.001, compared with the H/R + Ac2-26 group.

Hypoxia-reoxygenation (H/R) significantly increased AnxA1 protein expression at 2 h and 4 h after H/R in A549 cells when compared with the control group. Ac2-26 attenuated increased AnxA1 protein expression by H/R. Ac2-26 significantly reduced the increase of phosphorylated NF-κB p65 in the nucleolus and the decrease of IκB-α at 2 h and 4 h after H/R in A549 cells (Figure 10A–D). Furthermore, Ac2-26 significantly decreased the levels of IL-8 in the H/R group (Figure 10E). In contrast, the protective effects of Ac2-26 were significantly attenuated by the addition of Boc2.

## 3. Discussion

The study results demonstrated that AnxA1 peptide Ac2-26 significantly attenuated increased lung edema, PAP, neutrophil infiltration, inflammatory cytokine production, oxidative stress, apoptosis, MAPK activation, IκB-α degradation, nuclear translocation of NF-κB, and tissue damage in acute lung injury induced by IR. In addition, Ac2-26 significantly induced MKP-1 expression. However, these improvements are attributed to FPR activation, as the protective effect of Ac2-26 was reduced by the nonselective FPR antagonist, Boc2. Moreover, Ac2-26 treatment had a similar inhibitory effect on the reactivity of A549 alveolar epithelial cells exposed to H/R, corroborating the in vivo results. Our results suggest a protective role of AnxA1 in IR-induced lung injury and provide pharmacological evidence that the activation of FPRs may be the underlying mechanism for lung protection by Ac2-26.

AnxA1 has emerged as a potent anti-inflammatory therapeutic agent in a variety of diseases based on its ability to decrease inflammation both in vitro and in vivo, as well as to provide tissue protection [2]. Following IR injury, there was a significant increase in AnxA1 in the lung tissue, similar to that shown in previous studies [6,10,11,12,13]. The increased expression of AnxA1 was accompanied by leukocytes infiltrating the damaged tissues of the myocardium, brain, lung, and kidney [6,10,11,12,13]. As previously reported, the increased AnxA1 expression was at least partly due to transmigrating neutrophils [6,10,12,13]. The increase was attenuated by the administration of the AnxA1 peptide Ac2-26 [2,6]. In neutrophils, the AnxA1 protein was transported to the cell surface after neutrophils adhered to the endothelial cells. When on the cell surface, AnxA1 lessened leukocyte extravasation [2,6]; however, the blockade of the AnxA1 receptor inhibited neutrophil-endothelial cell interactions [6]. Therefore, the reduction in AnxA1 expression by Ac2-26 could be related to the suppression of the inflammatory response following an IR insult.

IR-induced lung injury is characterized by a marked polymorphonuclear leukocyte (PMNs) infiltration in the lung interstitium and alveolar space [7]. These observations were semi-quantitatively confirmed by both increasing MPO-positive cells and the number of neutrophils in the lung, both of which were attenuated by Ac2-26 treatment. Our findings were consistent with those of previous reports where treatment with Ac2-26 in rats significantly reduced PMN recruitment and MPO activity in IR-injured hearts, intestines, and LPS-induced lung injuries [10,11,12]. In addition, Ac2-26 significantly reduced the production of TNF-α and CINC-1 in the BALF and suppressed protein carbonylation and peroxidation of membrane lipids in the lung. Previous studies have also demonstrated that the inflammatory cytokine, TNF-α, and chemokine macrophage inflammatory protein 1-α were significantly reduced in IR-injured hearts treated with AnxA1 [14]. These reductions were also coupled with the suppression of IL-8 production in the human epithelial cell line A549 subjected to H/R in this study. Moreover, Ac2-26 attenuated pulmonary vascular permeability as indicated by reduced K_f_, LWG, W/D, and LW/BW ratios, and protein levels in the BALF, as well as the limited alteration of the tight junction proteins (claudin-3 and occludin) in the alveolar walls. These data were comparable with those of other experiments revealing that Ac2-26 has the ability to mitigate pulmonary vascular permeability in intestinal IR- and LPS-induced lung injury [10,11].

NF-κB is an important transcriptional factor responsible for activating the production of various cytokines and chemokines during inflammation. NF-κB activity is tightly controlled by its inhibitor, IκB, binding with NF-κB in the cytoplasm. We have previously demonstrated that IR-induced acute lung injury promoted IκB degradation and NF-κB activation [15,16]. In this study, we showed that Ac2-26 effectively disrupted the activation of the NF-κB signaling pathway in rat lung exposed to IR. Suppressing NF-κB activity led to a reduction in the production of pro-inflammatory mediators such as TNF-α. Comparable with the observations in the lungs, Ac2-26 significantly inhibited IκBα degradation and, as a consequence, nuclear translocation of NF-κB p65, and the production of IL-8 in A549 cells exposed to H/R. This finding was also consistent with the study by Zhang et al. [17], which demonstrated that AnxA1 was directly bound to the NF-κB p65 subunit and inhibited its activation in vitro and in vivo, and that the AnxA1 peptide Ac2-26 also had similar actions. Therefore, the regulation of the NF-κB signaling pathway could be a possible mechanism of Ac2-26 action against ALI induced by IR.

Several investigations revealed that AnxA1 promoted neutrophil apoptosis and modulated apoptotic neutrophils clearance by macrophages [2,5]. In contrast to our study, Ac2-26 treatment significantly decreased activated caspase-3-immunolabeled cells and increased Bcl-2 protein expression in the lung tissue after IR injury. Furthermore, it has been reported that TNF-α, one of the major mediators of ALI, can initiate the apoptotic cascade through the death receptor/caspase pathway [18]. As Ac2-26 attenuated TNF-α production in this study, it is reasonable to declare that Ac2-26, at least in part, inhibited apoptosis in ALI via an indirect manner. Whether Ac2-26 has a direct role in protecting neutrophils from apoptosis during ALI requires further investigation.

MAPKs are comprised of three major families: p38, ERK, and JNK protein kinases that modulate multiple cellular events and the production of inflammatory mediators. Abnormal activation of MAPK signaling results in a series of inflammatory cascades [19]. Previous studies have shown that IR, hemorrhagic shock, and LPS-induced lung injury increased the phosphorylation of p38, ERK, and JNK in the lung tissue [20,21,22]. These investigations also showed that the inhibition of p38, ERK, or JNK MAPK effectively suppressed LPS- or peritonitis-induced lung inflammation. In this study, Ac2-26 inhibited IR-induced activation of MAPKs. Similarly, treatment with the Tat-Anx-1 protein in an asthma mouse model markedly reduced the phosphorylation of ERK, p38, and JNK induced by ovalbumin, and this finding correlated with decreased levels of inflammatory cytokines [23]. Furthermore, the Tat-Anx-1 protein transfected into RAW 264.7 cells significantly reduced the LPS-induced production of inflammatory cytokines by blocking both NF-κB and MAPKs activity [24]. MKP-1 is a dual specificity protein phosphatase that is responsible for dephosphorylating all three MAPKs [25]. The increase of MKP-1 expression correlated with a reduction in the JNK, ERK, and p38 MAPK activities as a key negative regulator of inflammation [19]. Yang et al. reported that endogenous AnxA1 inhibited MAPK activation via the upregulation of MKP-1 in cultured fibroblasts [26]. Therefore, upregulation of MKP-1 may be an important mechanism for AnxA1 to limit the inflammatory response. The present study revealed that Ac2-26 increased the expression of MKP-1 protein, which resulted in the suppression of MAPK signaling. This subsequently stopped the vicious cycle of widespread inflammation in the IR-induced ALI. In our study, Boc2, an FPR1/FPR2 antagonist, abolished the beneficial effect of Ac2-26 in IR-induced ALI, which suggests that Ac2-26 worked via the FPR. Comparable findings were reported in murine experimental peritonitis and endotoxin-induced uveitis rodent models, where the anti-inflammatory effects of Ac2-26 were attenuated by Boc2 [27,28]. Moreover, Ac2-26 improved indomethacin-induced gastric ulcer healing, whereas Boc2 impaired healing [29]. Three FPRs exist in humans including FPR1, FPR2, and FPR3, which have important roles in the modulation of inflammatory responses and host defense [2]. AnxA1 interacts with only FPR2. In contrast, the mimetic peptide agonist Ac2-26 binds to both FPR1 and FPR2 [2]. In models of air-pouch and zymosan-induced peritonitis, the anti-migratory properties of AnxA1 and Ac2-26 were attenuated in Fpr2^−/−^ mice [30]. Delivery of the anti-inflammatory signal through Fpr2 was also supported by the experiment where Fpr2^−/−^ mice exhibited a striking exacerbation and prolongation of K/B × N serum transfer arthritis [30]. Previous studies showed that these FPR2 actions of Ac2-26 limited heart IR injury in vivo and in vitro, evidenced by reduced infarct size and cardiac myocyte injury [12,31]. Recently, the protective actions of Ac2-26 on the recovery of left ventricular function in the adult male rat heart were inhibited by the FPR1 antagonist [32]. However, the individual roles of FPR1 and FPR2 in IR-induced ALI remain to be elucidated.

IR-induced ALI is the major cause of primary graft dysfunction in the early stages after lung transplantation. The morbidity and mortality associated with IR-induced ALI is still high [7]. In our study, as Ac2-26 was demonstrated to have a protective effect in IR-induced ALI, the results could foster speculation and further research into its potential clinical effectiveness in planned lung transplantation and unplanned IR injury, such as traumatic hemorrhagic shock, resuscitation of cardiac arrest, and pulmonary embolism.

## 4. Material and Methods

### 4.1. Isolated Perfused Lung Model in Rats

Sprague-Dawley rats (male, 350 ± 20 g) were used in this study and cared for in accordance with the National Institutes of Health guidelines. The experimental protocol (approve number: IACUC-14-011, 5 February 2014) was approved by the Institutional Animal Care and Use Committee of the National Defense Medical Center. The isolated and perfused rat lungs were prepared using methods previously described in [16]. In brief, a tracheostomy was performed and the rat was ventilated with humidified air containing 5% CO_2_ accompanying with a 1-cm H_2_O positive end-expiratory pressure. The ventilator was set to deliver a tidal volume of 3 mL and 60 breaths/min. After a median sternotomy, the right ventricle was injected with heparin (1 U/g of BW), and 10 mL blood was collected by cardiac puncture. A cannula was inserted into the pulmonary artery and the other wide-bore cannula into the left ventricle. The pulmonary venous pressure (PVP), and the PAP were continuously recorded from the side arm of the cannula. The isolated lung was perfused with 10 mL physiological salt solution (119 mM NaCl, 4.7 mM KCl, 1.17 mM MgSO_4_, 22.6 mM NaHCO_3_, 1.18 mM KH_2_PO_4_, 1.6 mM CaCl_2_, 5.5 mM glucose, and 50 mM sucrose) containing 4% bovine serum albumin. The 10-mL sample of collected blood was added to the perfusate as the “half-blood” solution before recirculation. A roller pump was used to provide the constant flow rate, which was maintained at 8 mL/min. The isolated perfused lung in situ was placed on an electronic balance to record real-time lung weight changes.

### 4.2. Vascular Filtration Coefficient

The K_f_ was calculated from the change in LW caused by the elevation of venous pressure as previously described. K_f_ was defined as the *y*-intercept of the plot (in g·min^−1^) divided by the PVP (10 cm H_2_O) and LW, and expressed in whole units of g·min^−1^ cm H_2_O^−1^ × 100 g in [9,15].

### 4.3. Lung Weight/Body Weight (LW/BW) and Wet/Dry (W/D) Weight Ratios

The LW/BW and W/D weight ratios were determined as previously described in [15,16].

### 4.4. Assessment of Protein Concentration, CINC-1 and TNF-α Levels in BALF

The protein, TNF-α, and CINC-1 concentrations in the BALF were determined as previously described in [15,16].

### 4.5. Protein Carbonyl Content and MDA Level in Lung Tissue

The methods for measuring the MDA level and protein carbonyl content were described elsewhere in [15,16]. The levels of MDA and carbonyl contents were expressed as nmol/mg protein and nmol carbonyl/mg protein, respectively [16].

### 4.6. Western Blotting

Lung and cell culture protein lysates (30 μg/lane) were separated by 10–12% sodium dodecyl sulfate-polyacrylamide gel electrophoresis, and immunoblots were developed as previously described in [16]. The blots were probed with one of the following antibodies: anti-AnxA1 (1:1000, Bioss Inc., Woburn, MA, USA), anti-Bcl-2, anti-lamin B1 (1:200, Santa Cruz Biotechnology, Dallas, TX, USA), anti-NF-κB p65, anti-phospho-NF-κB p65, anti-IκB-α, anti-ERK1/2, anti-phosho-ERK1/2, anti-JNK, anti-phospho-JNK, anti-p38, anti-phospho-p38, anti-MKP-1 (1:1000, Cell Signaling Technology, Danvers, MA, USA), anti-TATA (1:1000, Abcam, Cambridge, MA, USA), or β-actin (1:10,000, Sigma Chemical Company, St. Louis, MO, USA). The data were presented as the relative ratio of the target protein to the reference protein. The relative ratio of the target protein of the control group was arbitrarily expressed as 1.

### 4.7. Immunohistochemical Analyses

Immunohistochemical staining to identify MPO and caspase-3 was performed as previously described in [16]. In brief, formalin-fixed paraffin lung tissue sections were de-paraffinized and pretreated for antigen retrieval. The endogenous peroxidase was quenched using 3% H_2_O_2_ and 100% methanol for 15 min. The lung sections were immunostained with the rabbit polyclonal antibody to MPO (1:100, Cell Signaling Technology) and rabbit polyclonal anti-caspase-3 antibody (1:200; Cell Signaling Technology). The slides were then incubated with rat-specific horseradish peroxidase-conjugated secondary antibody (Nichirei Corporation, Tokyo, Japan) for 30 min after being washed twice with phosphate-buffered saline (PBS). The horseradish peroxidase was visualized after a chromogenic reaction with diaminobenzidine for 3 min, and the lung tissue sections were counterstained with hematoxylin.

### 4.8. Histopathology

The numbers of polymorphonuclear neutrophils and lung injury score in the lung tissue were analyzed as previously described in [15,16]. Briefly, lung tissues were fixed, sectioned, and stained with hematoxylin and eosin. Morphologic examinations were performed using light microscopy. A minimum of 10 randomly selected fields was examined for neutrophil infiltration in the airspace or vessel wall, as well as for thickening of the alveolar wall. Next, lung damage was scored using a four-point scale: none (0), mild (1), moderate (2), or severe (3) lung injury by two pathologists blinded to the experimental condition. The resulting two scores were summed to represent the lung injury score.

### 4.9. Immunofluorescence Staining for Claudin-3, Occludin, and ZO-1

Immunofluorescence staining was performed as previously described in [33]. Rabbit polyclonal antibodies to claudin-3 and occludin (diluted 1:200, Invitrogen, Carlsbad, CA, USA) and mouse monoclonal antibody to ZO-1 (diluted 1:200, Invitrogen, Carlsbad, CA, USA) were used for immunofluorescent labeling. The labeled ZO-1 antibody was incubated for 30 min with goat anti-mouse IgG-fluorescein isothiocyanate (green) (diluted 1:200; Santa Cruz Biotechnology) at room temperature. Dylight 633 (red) goat anti-rabbit-IgG (Invitrogen) at 1:200 dilution was used as the secondary antibody to the labeled claudin-3 and occludin antibodies. Images were collected using a fluorescence microscope (Leica DM 2500, Wetzlar, Germany).

### 4.10. Experimental Design

The rat lungs were randomly assigned to receive PBS (control, *n* = 6), IR, IR with different doses of Ac2-26 (0.1 mg, 0.5 mg, or 1 mg/kg BW, *n* = 6 per group), and IR + Boc2 + Ac2-26 (1 mg/kg BW) (*n* = 6 per group). Ac2-26 (Ac-AMVSEFLKQAWFIENEEQEYVQTVK, Sanofi Aventis Company, St. Paul, MN, USA) was dissolved in 1 mL PBS and added to the reservoir (containing 20 mL of perfusate) for 10 min before lung ischemia. Boc2 (50 μg per rat), the FPR antagonist (ICN Pharmaceuticals, Basingstoke, UK), in 1 mL saline containing 1% dimethyl sulfoxide, was intraperitoneally injected into each animal 30 min before surgery. The doses of Ac2-26 and Boc2 in this study were chosen based on previous investigations in [12,28]. The rat lungs in the IR group were deflated by stopping ventilation and perfusion for 40 min to induce ischemia. Next, perfusion and ventilation were restored and persisted for 60 min.

### 4.11. Cell Culture and Induction of H/R

Human type II alveolar epithelial cells (A549) were obtained from the Food Industry Research and Development Institute (BCRC 60074, Hsinchu, Taiwan) and maintained in F-12K medium (Hyclone, Logan, UT, USA) containing 10% fetal bovine serum (Hyclone), penicillin, and streptomycin in a humidified atmosphere of 5% CO_2_–95% room air. A549 cells were subjected to 24 h of hypoxia (1% O_2_–5% CO_2_–94% N_2_) followed by 4 h of reoxygenation (5% CO_2_–95% room air) at 37 °C. Cells were pretreated with vehicle, Ac2-26 (0.3 μM), or Ac2-26 (0.3 μM) and Boc2 (10 μM) [32]. The control group was kept in the reoxygenated state without hypoxic stimulus. The human IL-8 ELISA kit (R&D, Inc., Minneapolis, MN, USA) was used for the quantitative measurement of IL-8 in the collected supernatant.

### 4.12. Data Analysis

We used GraphPad Prism 5 statistical software (GraphPad Software, San Diego, CA, USA) for the data analysis. Data were represented by means ± standard deviation. The comparisons among the groups were analyzed using a one-way analysis of variance (ANOVA) followed by a post-hoc Bonferroni test to evaluate the difference between each of the treatment and control groups. The lung weight gain and PAP between groups were compared by a two-way ANOVA analysis with repeated measurements followed by a post-hoc Bonferroni test. A value of *p* < 0.05 was defined as significant. 

## 5. Conclusions

In summary, we demonstrated that Ac2-26 attenuated IR-induced lung injury. However, the protective effect of Ac2-26 was mitigated by the presence of Boc2, a nonselective FPR inhibitor. Targeting the AnxA1 signaling pathway may provide a novel approach for the treatment of IR-induced lung injury.

## Supplementary Materials

Supplementary materials can be found at www.mdpi.com/1422-0067/18/8/1771/s1.
