# Correction: Wen-I Liao, et al. Ac2-26, an Annexin A1 Peptide, Attenuates Ischemia-Reperfusion-Induced Acute Lung Injury. *Int. J. Mol. Sci.* 2017, *18*, 1771

**DOI:** 10.3390/ijms19020526

**Published:** 2018-02-09

**Authors:** Wen-I Liao, Shu-Yu Wu, Geng-Chin Wu, Hsin-Ping Pao, Shih-En Tang, Kun-Lun Huang, Shi-Jye Chu

**Affiliations:** 1 The Graduate Institute of Medical Sciences, National Defense Medical Center, Taipei 114, Taiwan; qqww0139@yahoo.com.tw (W.-I.L.); simple5252@hotmail.com (H.-P.P.); 2 Department of Emergency Medicine, Tri-Service General Hospital, National Defense Medical Center, Taipei 114, Taiwan; 3 Institute of Aerospace and Undersea Medicine, National Defense Medical Center, Taipei 114, Taiwan; shuyu0321@gmail.com; 4 Department of Internal Medicine, Taoyuan Armed Forces General Hospital, Taoyuan 325, Taiwan; medicine804h@yahoo.com.tw; 5 Department of Internal Medicine, Tri-Service General Hospital, National Defense Medical Center, Taipei 114, Taiwan; t4n000@yahoo.com.tw

The authors would like to make a correction to their published paper [1].

There was a mistake in the original version of the article in Figure 10A (page 10). The arrangements of bands between groups in the AnxA1 panel were scrambled. The authors have corrected the error as shown in the figure below. The rest of the manuscript and the figure legend do not need to be changed. The authors would like to apologize for any inconvenience caused.

Please replace this figure:


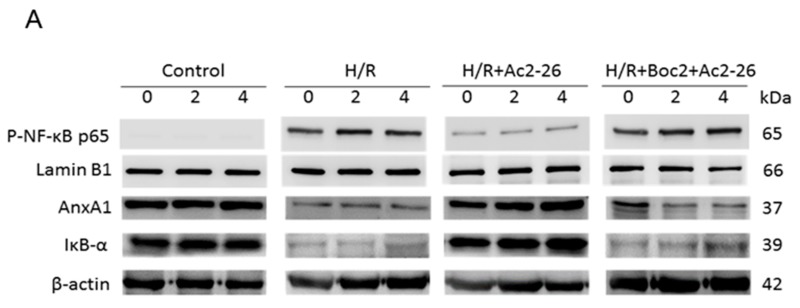
 with the following:


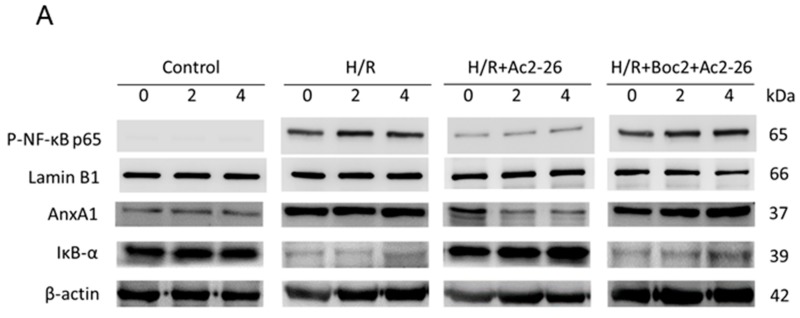


The changes do not affect the scientific results. The manuscript will be updated and the original will remain online on the article webpage, with a reference to this Correction.
