# Supplementary material for: Ac2-26, an Annexin A1 Peptide, Attenuates Ischemia-Reperfusion-Induced Acute Lung Injury
Source: Int J Mol Sci. 2017 Aug 15;18(8):1771. doi: 10.3390/ijms18081771 (PMC5578160; doi:10.3390/ijms18081771)
Supplement: Supplementary file 1 [file ijms-18-01771-s001.pdf]

Supplementary Material: Ac2-26, an Annexin A1 Peptide, Attenuates Ischemia/Reperfusion–Induced Acute Lung Injury

Wen-I Liao, Shu-Yu Wu, Geng-Chin Wu, Hsin-Ping Pao, Shih-En Tang, Kun-Lun Huang, and Shi-Jye Chu

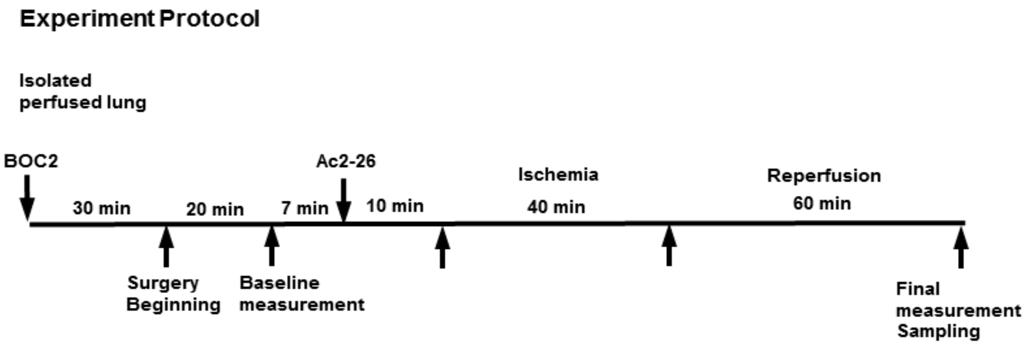

Figure S1. Experimental Protocol.

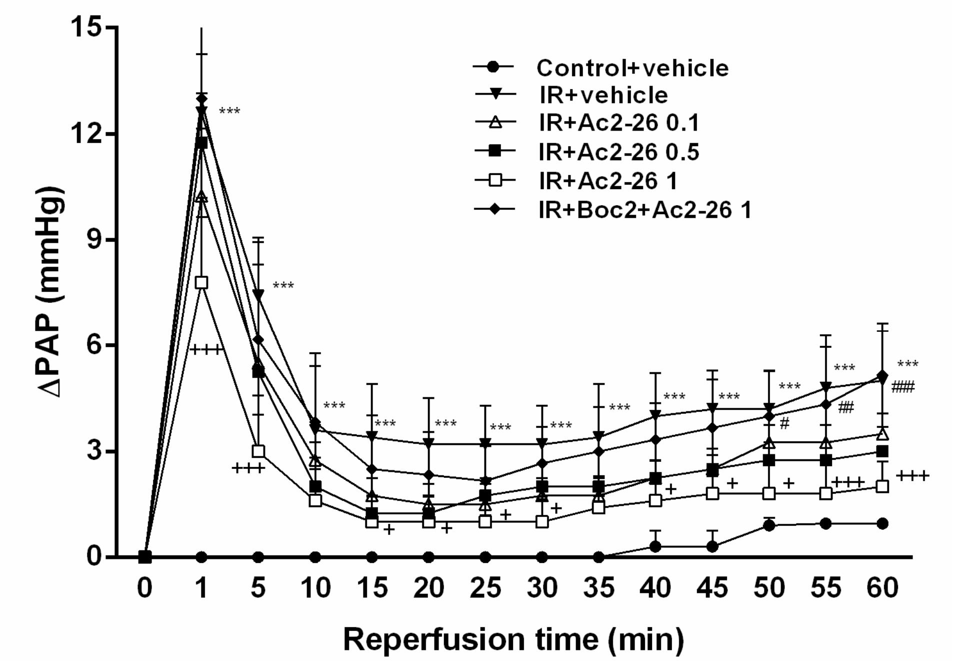

Figure S2. Effect of Ac2-26 and Boc2 on pulmonary artery pressure (ΔPAP). PAP increased significantly in the ischemia-reperfusion (IR) group. The increase in PAP was attenuated significantly by treatment with Ac2-26, but not when Boc2 was added. Data are expressed as mean ± SD (*n* = 6 per group). *** $p < 0.001$, compared with the control group; $^{+}$ $p < 0.05$, $^{++}$ $p < 0.01$, $^{+++}$ $p < 0.001$, compared with the IR group; $^{\#}$ $p < 0.05$, $^{\#\#}$ $p < 0.01$, $^{\#\#\#}$ $p < 0.001$, compared with the IR-AC2-26 1 group.
